# Programmed death-ligand 1 expression and overall survival in Thai patients with gastric cancer

**DOI:** 10.1038/s41598-023-34434-y

**Published:** 2023-05-04

**Authors:** Taned Chitapanarux, Pawut Gumrai, Sarawut Kongkarnka, Komson Wannasai, Nirush Lertprasertsuke

**Affiliations:** 1 Department of Internal Medicine, Faculty of Medicine, Chiang Mai University, Chiang Mai, 50200 Thailand; 2 Northern Thai Research Group of Radiation Oncology (NTRG-RO), Faculty of Medicine, Chiang Mai University, Chiang Mai, Thailand; 3 Department of Pathology, Faculty of Medicine, Chiang Mai University, Chiang Mai, Thailand

**Keywords:** Cancer, Biomarkers, Gastroenterology

## Abstract

Programmed death-ligand 1 (PD-L1) expression has now been implicated in gastric cancer (GC). This study was conducted to determine the impact of clinicopathological characteristics on PD-L1 expression and its association with survival in GC patients receiving standard-of-care. In total, 268 GC patients receiving upfront surgery were enrolled at Chiang Mai University Hospital. PD-L1 expression was assayed by immunohistochemistry staining using the Dako 22C3 pharmDx. The rates of PD-L1 positivity by combined positive score (CPS) at a cutoff value of 1 and 5 were 22% and 7%. PD-L1 positivity was significantly higher in patients younger than 55 than those older than 55 (32.6% vs. 16.5%, *p* = 0.003; 11.6% vs. 4.4%, *p* = 0.027). PD-L1 positivity was observed more frequently in GC with metastases than without (25.2% vs. 17.1%, *p* = 0.112; 7.2% vs. 6.7%, *p* = 0.673). Patients with PD-L1 positive had a significantly shorter median overall survival than those with PD-L1 negative (32.7 vs. 41.6 months, *p* = 0.042, 27.6 vs. 40.8 months, *p* = 0.038). In conclusion, PD-L1 expression has been associated with young age, short survival, and metastases, although unrelated to the tumor stage. For GC patients, PD-L1 testing is recommended, especially among young patients with metastases.

## Introduction

Gastric cancer (GC) is the fifth most common cancer worldwide, and gastric adenocarcinoma accounts for most GC cases^1^. Also, GC is the third leading cause of cancer-related deaths worldwide. The prognosis of GC remains poor, especially in advanced stages, even with multidisciplinary therapies that improve treatment outcomes^2^. One of the most challenging problems in the clinical treatment of GC is that only a part of GC patients benefits from traditional chemical treatment strategies, indicating that other considerations, such as the human immune reaction, also affect the clinical outcome.

Immunotherapeutic agents targeting immunosuppressive proteins have been recognized as potential treatments for cancer due to their favorable curative effect and improved survival time. Among these agents, anti-programmed death protein-1/ligand 1 (PD-1/PD-L1) antibodies are considered the most exciting advancements in cancer immunotherapy^3^. PD-1/PD-L1 checkpoint inhibitors have shown promising results in treating many types of cancer, including recurrent locally advanced or metastatic GC^4,5^. Furthermore, the PD-L1 protein expression in viable cancer cells determined by immunohistochemistry (IHC) correlates with the therapeutic effect of immune checkpoint inhibitors. The Food and Drug Administration (FDA) recently approved PD-L1 IHC as a predictive biomarker for the anti-PD-L1 response for some solid tumors, including GC^6^. In addition, the combined positive score (CPS) is validated as a robust and reproducible method to score PD-L1 protein expression for GC patients treated with pembrolizumab^7,8^. In CheckMate-649, compared with chemotherapy, the survival benefits of first-line nivolumab combined with chemotherapy increased with the CPS cutoff value^9^. There are many studies on the clinicopathological and prognostic significance of PD-L1 expression in GC. However, the PD-L1 expression in the Thai population with GC has yet to be evaluated. Furthermore, there is limited data about the prognostic significance of PD-L1 expression among GC patients receiving standard-of-care. Therefore, the primary objective of this study was to examine the rate of PD-L1 expression in Thai patients with GC. Other purposes were to characterize PD-L1 expression and its association with clinicopathological features and the survival of patients with GC.

## Materials and methods

### Patients and data collection

We retrospectively enrolled GC patients who underwent upfront surgery at Chiang Mai University Hospital, Thailand, from January 1, 2018, to December 31, 2021. All patients were diagnosed with gastric adenocarcinoma by the pathological results of H&E staining specimens. Two experienced pathologists reviewed all cases and confirmed the histological diagnoses without discrepancy. The exclusion criteria were those who received neoadjuvant therapy and those with tumors that were not gastric adenocarcinoma. In addition, patients who died postoperatively due to surgical-related complications were also excluded. Clinical characteristics, including age, sex, tumor location, tumor size, pathologic stage (pTNM), histologic type based on Lauren classification, lymph node status, and vascular invasion, were obtained from hospital medical records and extracted from Chiang Mai University Hospital's electronic database. All patients received standard-of-care for GC therapy. Telephone interviews and medical records were used as follow-up procedures. This study was registered at thaiclinicaltrials.org (number TCTR20221031001). Written informed consent was obtained from all participants included in the study. The study was approved by the Research Ethics Committee Faculty of Medicine, Chiang Mai University (MED-2562–06300) and conducted in accordance with the Helsinki Declaration and its later amendments or comparable ethical standards.

### Immunohistochemistry (IHC) staining and evaluation

IHC was performed on 4 -µm-thick tissue sections using an automated IHC Stainer (Ventana, Tucson, AZ, USA). The assessment of PD-L1 protein expression in GC is a qualitative IHC assay that uses anti-PD-L1 antibodies (Dako, 22C3) to detect PD-L1 protein in formalin-fixed, paraffin-embedded tissues from gastric adenocarcinomas. A minimum of 100 tumor cells must be present in the PD-L1 stained slide for the specimen to be considered adequate for PD-L1 evaluation. Expression of PD-L1 was reported as CPS, defined as the total number of PD-L1 positive cells (lymphocytes, macrophages, and tumor cells) divided by the total number of viable tumor cells^10^. The CPS ≥ 1 and ≥ 5 were chosen to define PD-L1 positive. A monoclonal antibody against Latent Membrane Protein (LMP)-1 (CS1-4; Dako, Glostrup, Denmark) was used to detect EBV-specific protein to identify EBV status for GC with CPS ≥ 1. IHC for LMP-1 was done according to the method previously described^11^. Brown granular cytoplasmic and membrane staining was interpreted as positive for EBV LMP-1, whereas bluish staining of the cytoplasm and membrane was interpreted as negative for EBV LMP-1. A positive control included a tissue known to have EBV infection, whereas, for negative controls, the test antibody was omitted and replaced by phosphate-buffered saline.

### Statistical analysis

All statistical analyses were performed using Stata software, version 15.1 MP (Stata Corporation, College Station, Texas, USA). Data for categorical variables was shown by frequency and percentage. As appropriate, the comparison between PD-L1 expression and clinicopathological features of GC was analyzed using the Chi-squared test or Fisher's exact test. Overall survival was defined as the time from the initial diagnosis to death by any cause or last follow-up. The relationship between PD-L1 expression and overall survival was analyzed using the Kaplan–Meier method and log-rank tests, with PD-L1 negative as the reference. A two-tailed *p* value < 0.05 was considered statistically significant.

### Ethical approval

The study conformed to the principles of the Declaration of Helsinki and Good Clinical Practice Guidelines. It was approved by the Research Ethics Committee Faculty of Medicine, Chiang Mai University (MED-2562–06300).

### Consent to participate

Patients received written and oral information on the study and gave their consent to participate and use their medical data for research purposes.

## Results

### Clinicopathological characteristics

A total of 268 patients with GC were included in this study. None of the patients received chemotherapy or radiation before surgery. There were 132 (49%) males and 136 (51%) females with a mean age of 59.0 ± 10.2 years (range 37–87 years) at diagnosis. Tumor location was in the lower part of the stomach for 51%, the middle part for 28%, and the upper part for 21%. A tumor diameter of less than 5 cm accounted for 49% of patients, whereas a tumor diameter of more than 5 cm accounted for 51%. Lauren classification was diffuse for 55% and intestinal type for 45%. According to the pTNM classification, the disease was stage I, II, III, IV, and undetermined at 3%, 12%, 25%, 52%, and 8%, respectively. Lymph node metastasis was 44%, and vascular invasion was 23%. More detailed clinicopathological characteristics are summarized in Table 1.

**Table 1:** Clinicopathological features of gastric cancer.

Characteristics		n (%)
Sex	Male	132 (49.3)
	Female	136 (50.7)
Age (yr.)	< 40	22 (8.2)
	40–60	114 (42.5)
	60–80	122 (45.5)
	> 80	10 (3.7)
Stage	I	8 (3.0)
	II	32 (11.9)
	III	66 (24.6)
	IV	140 (52.2)
	Undetermined	22 (8.2)
Lauren classification	Intestinal	120 (44.8)
	Diffuse	148 (55.2)
Tumor location	Upper part	56 (20.9)
	Middle part	76 (28.4)
	Lower part	136 (50.7)
Tumor diameter	≤ 5 cm	132 (49.3)
	≥ 5 cm	136 (50.7)
Lymphatic invasion		118 (44.0)
Vascular invasion		62 (23.1)

### Correlation between PD-L1 expression and clinicopathological characteristics

The positive rates of CPS with a cutoff value of 1 and 5 were 22% (58/268) and 7% (18/268), respectively. The relationship between clinicopathological characteristics and PD-L1 expression are summarized in Table 2. Among all clinicopathological characteristics, PD-L1, CPS ≥ 1, and PD-L1, CPS ≥ 5 were significantly higher in patients younger than 55 than those older than 55 (32.6% vs. 16.5%, *p* = 0.003; 11.6% vs. 4.4%, *p* = 0.027). PD-L1 expression is age-related in patients with GC. PD-L1 positivity was observed more frequently in GC with metastases than without (25.2% vs. 17.1%, *p* = 0.112; 7.2% vs. 6.7%, *p* = 0.673). However, no significant correlations were observed between PD-L1 expression and gender, tumor location, tumor diameter, pTNM stage, Lauren classification, lymphatic and vascular invasion, or metastases. Among GC with CPS ≥ 1, 30 (52%) of 58 patients had IHC positive for EBV LMP-1.

**Table 2:** PD-L1 expression by study subgroup.

n (%)	CPS ≥ 1	CPS < 1	*p* value	CPS ≥ 5	CPS < 5	*p* value
Sex						
Male	28 (21.2)	104 (78.8)	0.867	8 (6.7)	124 (93.3)	0.673
Female	30 (22.1)	106 (77.9)		10 (7.4)	126 (92.6)	
Age						
≤ 55	28 (32.6)	58 (67.4)	**0.003**	10 (11.6)	76 (88.4)	**0.027**
> 55	30 (16.5)	152 (83.5)		8 (4.4)	174 (95.6)	
Stage						
Early (I & II)	8 (20.0)	32 (80.0)	0.837	4 (10.0)	36 (90.0)	0.476
Advanced (III & IV)	44 (21.4)	162 (78.6)		14 (6.8)	192 (93.2)	
Lauren classification						
Intestinal	30 (25.0)	90 (75.0)	0.096	13 (10.8)	107 (89.2)	0.057
Diffuse	16 (13.7)	101 (86.3)		5 (4.3)	112 (95.7)	
Tumor location						
Lower part	26 (19.1)	110 (80.9)	0.308	8 (5.9)	128 (94.1)	0.579
Other parts	32 (24.3)	100 (75.7)		10 (7.6)	122 (92.4)	
Tumor diameter						
≤ 5 cm	28 (21.1)	105 (78.9)	0.816	9 (6.8)	124 (93.2)	0.974
> 5 cm	30 (22.2)	105 (77.8)		9 (6.7)	126 (93.3)	
Lymphatic invasion						
Positive	20 (16.9)	98 (83.1)	0.098	8 (6.8)	110 (93.2)	0.971
Negative	38 (25.3)	112 (74.7)		10 (6.7)	140 (93.3)	
Vascular invasion						
Positive	12 (19.4)	50 (80.6)	0.618	5 (8.1)	57 (91.9)	0.629
Negative	46 (22.3)	160 (77.7)		13 (6.3)	193 (93.7)	
Metastases						
Positive	38 (25.2)	113 (74.8)	0.112	11 (7.2)	140 (92.8)	0.673
Negative	20 (17.1)	97 (82.9)		7 (6.3)	110 (93.7)	

Statistically significant *p* values are in bold (*p* < 0.05).

### Expression of PD-L1 and clinical outcomes

**Figure 1:**
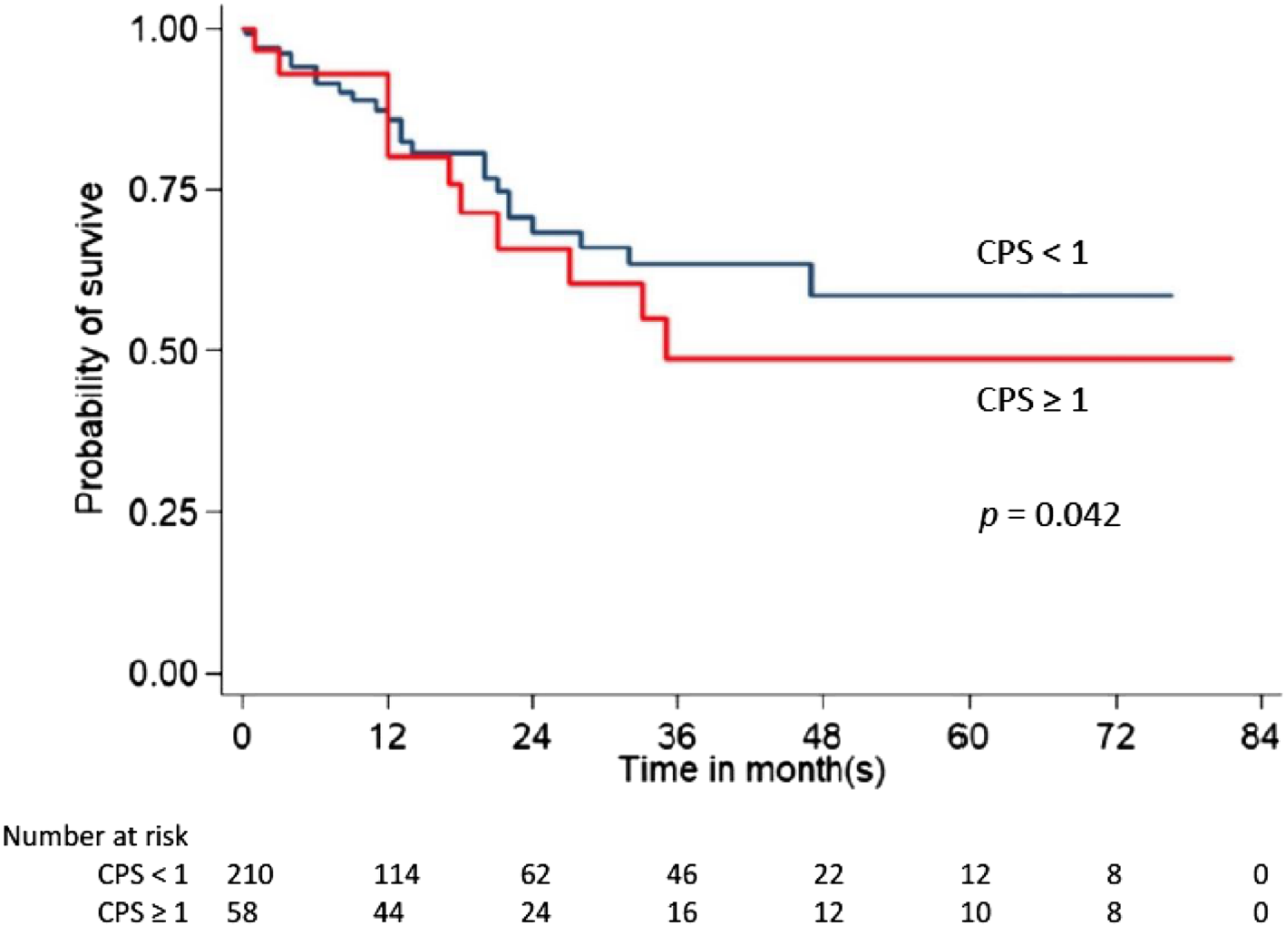
Kaplan–Meier analysis of CPS ≥ 1 and overall survival.

**Figure 2:**
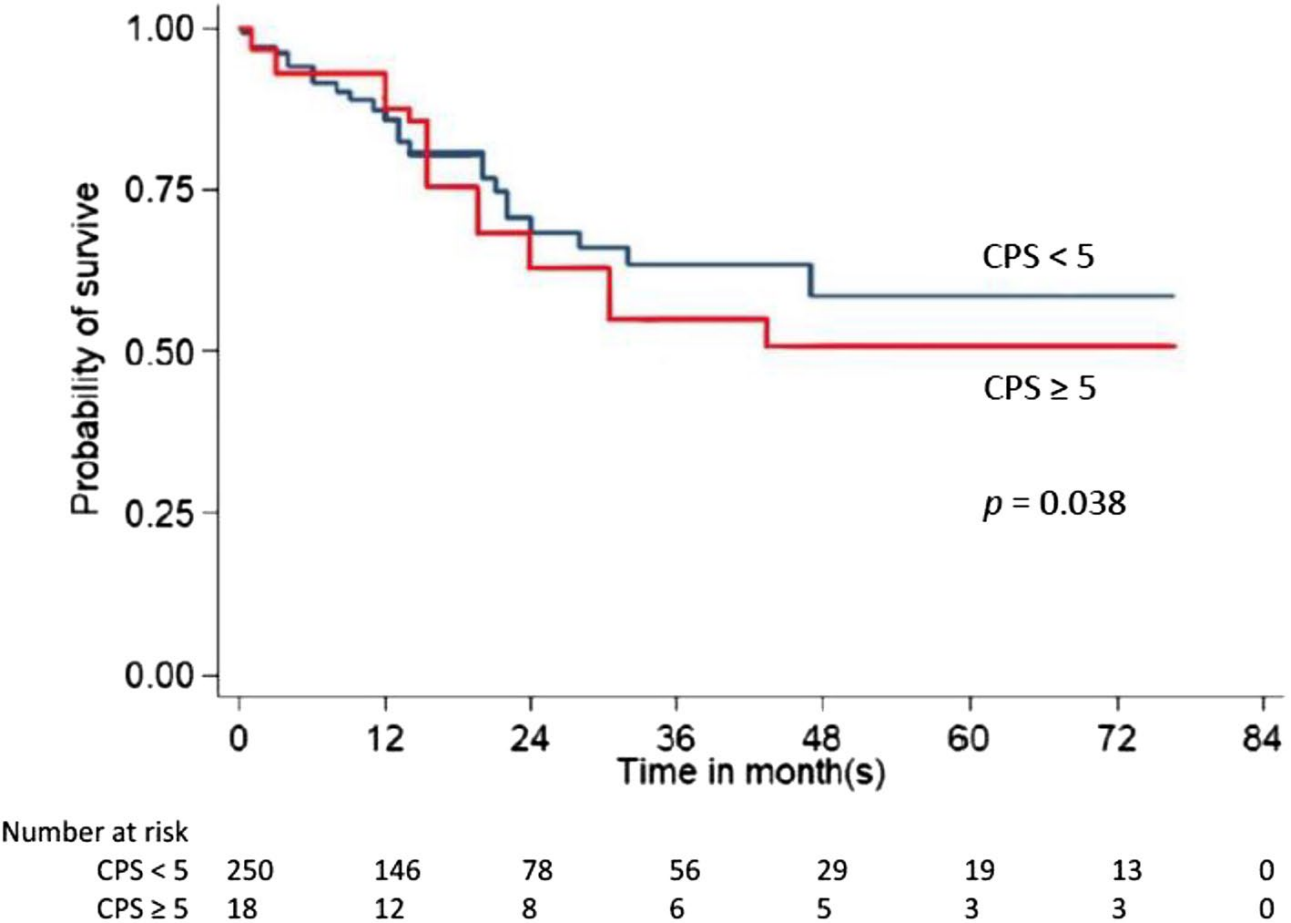
Kaplan–Meier analysis of CPS ≥ 5 and overall survival.

We investigated the prognostic significance of PD-L1 expression concerning overall survival. Based on CPS, overall survival is represented by the Kaplan–Meier curve in Figs. 1 and 2. Our study revealed that the median overall survival was significantly shorter in patients with PD-L1 positive (CPS ≥ 1 and ≥ 5) than in those with PD-L1 negative (32.7 vs. 41.6 months, *p* = 0.042, 27.6 vs. 40.8 months, *p* = 0.038).

## Discussion

PD-1/PD-L1 immune checkpoint inhibitors are now approved for treating patients with advanced GC^12,13^. PD-L1 expression, evaluated by IHC, is accepted as a predictive biomarker for the effectiveness of PD-1/PD-L1 inhibitors^14^. This present study is the first evaluation of PD-L1 expression in Thai patients with GC. The prevalence of PD-L1 expression with a CPS cutoff value of 1 and 5 in patients with GC was 22% and 7%, respectively. Patients with PD-L1 positive were typically younger and had significantly shorter survival than those with PD-L1 negative. PD-L1 expression is common in GC patients with metastases. PD-L1 overexpression appears to be an unfavorable prognostic factor in GC.

Our study describes findings from the clinical audit of PD-L1 expression in GC, providing the first insight into the rate of PD-L1 positivity in gastric adenocarcinoma in Thailand. Based on 268 cases of GC analyzed for PD-L1 expression, patients with PD-L1, CPS ≥ 1, and PD-L1, CPS ≥ 5 accounted for 22% and 7% of participants, respectively. The rate of PD-L1 positivity was lower than that reported in the literature from different populations (43% to 63%)^15–19^. This low expression rate of PD-L1 may be attributed to correlated factors, including a patient cohort, ethnic differences, different types of tumor samples or staging, IHC staining method, and positive cutoff levels for PD-L1 expression. Our study used the IHC 22C3 pharmDx, the only companion diagnostic assay approved by the FDA, at the CPS ≥ 1 and ≥ 5 cutoffs to assess the PD-L1 expression in GC^20^. Moreover, we used surgical resection samples to avoid intratumoral heterogenicity from biopsy specimens and for precise pathological staging. Although, our study found no statistically significant correlation between PD-L1 positivity and gender, pTNM stage, Lauren classification, tumor location, tumor size, lymphatic invasion, vascular invasion, or metastases. However, PD-L1, CPS ≥ 1 and PD-L1, CPS ≥ 5 had a statistically significant correlation with age lower than 55 (32.6% vs. 16.5%, *p* = 0.003; 11.6% vs. 4.4%, *p* = 0.027). Consistent with the previous report, PD-L1 expression was more common in young-onset than average-onset GC patients (31% vs. 3%, *p* < 0.05)^21^.

GC is an epithelial tumor associated with Epstein-Barr virus (EBV) infection confirmed by EBV type A and wild-type LMP1 variants in GC lesions in the Thai population^22^. Based on epidemiological data, 95% of adult Thais have immunity to EBV from childhood infection^23^. Thus, EBV-positive GC is found in younger patients more often than in EBV-negative gastric tumors^24^. More than half of the GC patients in our study have been infected with EBV. Likewise, in the previous studies from Brazil and Turkey, the positivity of EBV was 50% to 60% in gastric cancer tissues^25,26^. EBV induces intra- or peri-tumoral immune cell infiltration and shows genomic amplification of the chromosome 9 locus containing the genes encoding PD-L1^27^. In addition, EBV has upregulated expression levels of PD-L1 in cancer and immune cells^28^. Consequently, overexpression of PD-L1 is observed in young patients with EBV-associated GC^29,30^. Moreover, elderly patients have low levels of PD-L1 expression due to immune senescence caused by thymic involution and decreased synthesis of T cell progenitors from bone marrow^31^. These reasons explain the results of our study showing that PD-L1 positivity was more common in young Thai patients than in elderly patients with GC. We hypothesize that EBV plays a role in the pathogenesis of GC by enhancing PD-L1 expression and provides potentially relevant biomarkers for selecting patients who may derive more significant benefits from PD-1/PD-L1 checkpoint inhibitors, an emerging novel treatment option for GC.

The impact of PD-L1 expression on prognosis remains controversial in several malignancies^19,32–35^. In our study, PD-L1 positivity in Thai patients with GC was associated with poor prognosis and higher mortality, reducing the chances of overall survival. These findings are related to the PD-L1 positivity, which was more common in patients with metastases than without. Supporting our findings, a meta-analysis on GC patients revealed that PD-L1 positivity corresponded to a poor prognosis for overall survival^36,37^. Patients with PD-L1 expression should receive immunotherapy instead of standard-of-care for GC. Therefore, PD-L1 expression can be used as a reliable indicator for monitoring the clinical prognosis of GC patients.

However, there are certain limitations of this study. This study was a retrospective analysis that used archived tissue specimens from tissue blocks which likely influenced the amount of PD-L1 expression that may change over time. In addition, since this was a single-center study, selection bias may have existed. Given these limitations, it is probably improper to consider our results as a wholly accurate representation of the prevalence of PD-L1 expression in GC. A well-conducted prospective randomized multicenter trial can give us the exact prevalence of PD-L1 expression and its clinicopathological correlation with GC in Thailand. However, our study can provide insights for improving the selection of patients eligible for anti-PD-1/PD-L1 therapy.

## Conclusion

Accurate assessment of PD-L1 expression in GC in the Thai population provides valuable data unique to Thai patients and allows for the cost-effective management of cancer in this population. PD-L1 expression was evident in one-fourth of Thai patients with GC. Furthermore, the expression of PD-L1 has been associated with young age, short survival, and promoting metastases, although unrelated to the tumor stage. Therefore, PD-L1 testing is recommended, especially among young GC patients with metastases, to select patients eligible for anti-PD-1/PD-L1 therapy.

## Data Availability

Datasets analyzed during the current study are available from the corresponding author upon reasonable request.

## Abbreviations

CPS: Combined positive score
EBV: Epstein-Barr virus
GC: Gastric cancer
IHC: Immunohistochemistry
PD-1: Programmed death protein-1
PD-L1: Programmed death-ligand 1
